# Reports on the Hospitals of the United Kingdom

**Published:** 1909-10-09

**Authors:** Henry Burdett


### REPORTS
ON THE
### Hospitals of the United Kingdom.

By SIR HENRY BURDETT, K.C.B., K.C.V.O.

### II.—THE CHICHESTER GENERAL INFIRMARY.

### Designed 80 Years Ago and Good Still.

The buildings of this Infirmary have a special interest in the sense that they remain practically to-day, so far as the wards are concerned, as they were originally planned by the architect 80 years ago. This Infirmary may be regarded as probably the best specimen extant of the old "H" plan of construction. The reason for this distinction is probably twofold. First the buildings were more than large enough originally to provide for the requirements of the district which it serves, and secondly the managers for the time being have had the courage and wisdom to keep abreast of the times to the essential extent, without launching upon any large or excessive expenditure for buildings or appliances. Indeed, if there is any generous-minded person amongst the Governors, he or she might send the committee a cheque for £500 to defray the cost of new fittings and of the latest appliances in lieu of several which, though possibly sufficient, are not sightly or modern, or in harmony with the many good and attractive features of the Chichester Infirmary. Whilst dealing with the buildings it may be well to point out that the basement rooms have been made light and airy by excavations at an angle which makes them, for practical purposes, ground-floor rooms. This fact we commend to the modern architect, who is not always alive to the necessity of this precaution where a relatively small site renders it necessary to utilise the basement for day room space.

### A Zealous and Capable Administration.

Our visit of inspection was made without any intimation to the authorities, and everything bore its workaday aspect. It affords us very great pleasure to testify that throughout the establishment there was evidence that those immediately responsible for the administration are zealous and capable and well up to their work. It is the practice, for instance, to admit infants; yet the children's ward, containing probably from 18 to 20 cots, was in perfect order, and all the occupants were contentedly happy. Again, the dispensary—the dispenser is a woman—was the best kept, the cleanest and the most orderly, on the whole, that we have seen for a long time. The stores, linen, operation-theatre and wards were well kept and arranged. Indeed, the only doubtful point which struck us was our meeting with the large rice-puddings and some other food at a quarter to twelve, as they were proceeding from the kitchen to the wards, where we noticed no means for keeping the food hot. We were told the dinner-hour was 1 o'clock, not 12, in which case portions of the patients' dinners could not be served hot.

### An Isolation Ward Defect.

The general impression given to the visitor is a pleasant one, and we shall not be surprised to hear that the house-surgeon and other residents remain for years! The institution has a homelike air, is situated in pleasant surroundings, and its supporters have good reason to be proud of it. Structurally, the chimney to the Isolation Ward should be carried up to the same height as the chimneys in the main block, so as to make this ward habitable when a fire is required. We would suggest, further, that the linen cupboards should be ventilated.

### The Nursing Department.

The nursing department is under a matron, whom we were sorry to miss. The whole of the nurses, including upwards of 20 on the private staff, are accommodated on the top floor of the building, where each nurse is provided with a separate bedroom. Having regard to the size of the staff, we would suggest an additional bath-room containing a bath of a larger size than that at present in use. The furniture in the nurses' sitting-room is too limited, and more, and more comfortable chairs are essential owing to the number of the staff. The appurtenances of the nurses' dining-table exhibited a recognition of the claims which nurses have on the management of a hospital for reasonable comforts. The night nurses sleep in rooms which can be shut off, but the door is not always fastened when they are in bed, and the iron gratings over the heating apparatus in the long corridor of approach make the day traffic unusually noisy. This is a small point, but calls for immediate adjustment.

### The King's Wisdom—a Problem for the Governors.

The public has come to recognise and appreciate the care and consideration bestowed by King Edward VII. upon all matters which come before him. We gather that he recently made careful inquiries into the management of the Chichester Infirmary and was advised that it was satisfactory. His inquiries were due, we imagine, to a request made to him by the Governors that he would graciously permit the word "Royal" to be included in the title of the institution. King Edward's reply was, we understand, that the institution was in debt to the extent of some £2,000, and that to be "Royal" an institution must be without debt. Wherever a hint of this kind has been thrown out before in connection with a hospital in this country, it has been promptly acted upon. We imagine that the authorities of the Chichester Infirmary will not take King Edward's hint "sleeping," but that they will quit themselves like men by actively circulating the facts here recorded, ask for the support of the Governors of the Chichester Infirmary, raise the £2,000, and so worthily put this institution in a position to apply once more for the King's endorsement of its work. If the President and Committee take these steps in the course of October, we have no doubt that before Christmas the whole £2,000 can be easily raised, and that this attractive, well-ordered and excellent institution may then take a new lease of life and usefulness from January 1, 1910, as "The Royal West Sussex and Chichester Infirmary."

Some Suggestions.

We are surprised to find that this hospital does not keep its accounts upon the uniform system and so place its management upon an up-to-date basis. All the best administered hospitals in the country now adopt the uniform system, which has extended throughout the empire, for it places the managers in a position to compare their expenditure under all heads with that of other hospitals upon an identical basis. We note, too, that partners in the same firm are eligible to be appointed members of the medical staff, a rule which does not always work well in practice. It should be made a rule that where two partners are members of the same staff one must be a physician and the other a surgeon. In no case should one partner be eligible to take charge of all the beds allotted to members of his firm. In the present day, hospital work is much more responsible than ever before, and the requisite time to attend to all the in-patients admitted to his beds, and to all who attend on his out-patient days should be given regularly to the work by each member of the staff. When he fails to do this an honorary medical officer should resign.

**The Royal Hants County Hospital, Winchester** will be reported on next week.

